# Carnosine, Small but Mighty—Prospect of Use as Functional Ingredient for Functional Food Formulation

**DOI:** 10.3390/antiox10071037

**Published:** 2021-06-28

**Authors:** Ivana Jukić, Nikolina Kolobarić, Ana Stupin, Anita Matić, Nataša Kozina, Zrinka Mihaljević, Martina Mihalj, Petar Šušnjara, Marko Stupin, Željka Breškić Ćurić, Kristina Selthofer-Relatić, Aleksandar Kibel, Anamarija Lukinac, Luka Kolar, Gordana Kralik, Zlata Kralik, Aleksandar Széchenyi, Marija Jozanović, Olivera Galović, Martina Medvidović-Kosanović, Ines Drenjančević

**Affiliations:** 1 Department of Physiology and Immunology, Faculty of Medicine, Josip Juraj Strossmayer University of Osijek, J. Huttlera 4, HR-31000 Osijek, Croatia; ivana.jukic@mefos.hr (I.J.); nikolina.bilic.dujmusic@gmail.com (N.K.); ana.stupin@mefos.hr (A.S.); anitaa3006@gmail.com (A.M.); kozina.natasa@gmail.com (N.K.); zmihaljevic@mefos.hr (Z.M.); martina.mihalj@gmail.com (M.M.); psusnjara1@gmail.com (P.Š.); marko.stupin@gmail.com (M.S.); aleksandar_mf@yahoo.com (A.K.); 2 Scientific Center of Excellence for Personalized Health Care, Josip Juraj Strossmayer University of Osijek, Trg Svetog Trojstva 3, HR-31000 Osijek, Croatia; zeljka.breskic@gmail.com (Ž.B.Ć.); selthofer.relatic@gmail.com (K.S.-R.); lukinac28@gmail.com (A.L.); lukakolar.vu@gmail.com (L.K.); gkralik@fazos.hr (G.K.); zlata.kralik@fazos.hr (Z.K.); szealex@kemija.unios.hr (A.S.); mjozanovic@kemija.unios.hr (M.J.); ogalovic@kemija.unios.hr (O.G.); mmkosano@kemija.unios.hr (M.M.-K.); 3 Department of Pathophysiology, Physiology and Immunology, Faculty of Dental Medicine and Health, Josip Juraj Strossmayer University of Osijek, Cara Hadrijana 10E, HR-31000 Osijek, Croatia; 4 Department of Dermatology and Venereology, University Hospital Osijek, HR-31000 Osijek, Croatia; 5 Department for Cardiovascular Disease, University Hospital Osijek, J. Huttlera 4, HR-31000 Osijek, Croatia; 6 Department of Internal Medicine, General Hospital Vinkovci, Zvonarska 57, HR-32100 Vinkovci, Croatia; 7 Department for Internal Medicine, Faculty of Medicine, Josip Juraj Strossmayer University of Osijek, J. Huttlera 4, HR-31000 Osijek, Croatia; 8 Department of Rheumatology, Clinical Immunology and Allergology, Clinical Hospital Center Osijek, J. Huttlera 4, HR-31000 Osijek, Croatia; 9 Department of Internal Medicine, Vukovar General Hospital, HR-32000 Vukovar, Croatia; 10 Nutricin j.d.o.o. Darda, HR-31326 Darda, Croatia; 11 Department of Animal Production and Biotechnology, Faculty of Agrobiotechnical Sciences, Josip Juraj Strossmayer University of Osijek, Vladimira Preloga 1, HR-31000 Osijek, Croatia; 12 Department of Chemistry, Josip Juraj Strossmayer University of Osijek, Cara Hadrijana 8/A, HR-31000 Osijek, Croatia

**Keywords:** oxidative stress, antioxidants, functional food, carnosine

## Abstract

Carnosine is a dipeptide synthesized in the body from β-alanine and L-histidine. It is found in high concentrations in the brain, muscle, and gastrointestinal tissues of humans and is present in all vertebrates. Carnosine has a number of beneficial antioxidant properties. For example, carnosine scavenges reactive oxygen species (ROS) as well as alpha-beta unsaturated aldehydes created by peroxidation of fatty acid cell membranes during oxidative stress. Carnosine can oppose glycation, and it can chelate divalent metal ions. Carnosine alleviates diabetic nephropathy by protecting podocyte and mesangial cells, and can slow down aging. Its component, the amino acid beta-alanine, is particularly interesting as a dietary supplement for athletes because it increases muscle carnosine, and improves effectiveness of exercise and stimulation and contraction in muscles. Carnosine is widely used among athletes in the form of supplements, but rarely in the population of cardiovascular or diabetic patients. Much less is known, if any, about its potential use in enriched food. In the present review, we aimed to provide recent knowledge on carnosine properties and distribution, its metabolism (synthesis and degradation), and analytical methods for carnosine determination, since one of the difficulties is the measurement of carnosine concentration in human samples. Furthermore, the potential mechanisms of carnosine’s biological effects in musculature, metabolism and on immunomodulation are discussed. Finally, this review provides a section on carnosine supplementation in the form of functional food and potential health benefits and up to the present, neglected clinical use of carnosine.

## 1. Introduction

Carnosine is a dipeptide synthesized in the body from β-alanine and L-histidine. It was originally discovered in skeletal muscle [1] where it is present in larger amounts than in other tissues, but it is also found in high concentrations in the brain, heart, and gastrointestinal tissues of humans. It is an integral part of different tissues in all vertebrates, while plants do not contain it at all [2,3].

Although its physiological role has not been completely understood yet, carnosine is a non-enzymatic free-radical scavenger and a natural antioxidant [4] and has anti-inflammatory and neuroprotective properties [5,6]. Furthermore, its numerous beneficial effects have now been well established including pH-buffering activity [7], heavy metal chelating activity [8], and antiglycating activity [9,10]. Carnosine reduces lipid peroxidation, but also inhibits oxidative modification of protein exposed to hydroxyl radicals [11]. Furthermore, it improves antioxidant capacity [12].

Studies have shown that the chronic oral ingestion of β-alanine can substantially elevate (up to 80%) the carnosine content of human skeletal muscle, which is the major production and storage site for carnosine in the human body [13]. Furthermore, data indicate that carnosine has excellent potential for use as a natural antioxidant in processed foods [14], since it has been suggested to be absorbed intact through the intestine into the blood [15]. Under normal resting conditions, carnosine is low in plasma due to the large amount of carnosinase [16]. After meat digestion, the quantity of dietary carnosine available for intestinal absorption depends on its original concentration in food and also on its bioaccessibility [17]. For example, after beef consumption, carnosine is detected in plasma within 15 min, reaching a maximum after 3.5 h, whereas 5.5 h after carnosine consumption, carnosine concentrations fall below detectable serum carnosine values [18]. Since carnosine has several beneficial properties, evidence of its bioavailability suggests that it could be suitable for use as a functional food ingredient. The main differences between the ingestion of carnosine from food matrix vs. supplements (e.g., capsule) would be in its bioaccessibility and bioavailability. Indeed, the release and absorption rate of carnosine from meat products would be proportional to its original concentration in the food product and would depend on the gastrointestinal environment, while the absorption rate of carnosine upon ingestion of capsule supplements would be equal to the dose indicated on the label [19].

It is demonstrated that carnosine can prolong cell life and preserve cellular homeostasis [3]. Considering all beneficial properties of carnosine and its clinical use [20], carnosine can be recommended as a natural cure that has no side effects but is highly efficient [21]. Furthermore, natural dipeptide antioxidants (such as carnosine) are receiving increasing attention because of their noticeable potential as biopreservatives in food recent technology [22].

The aim of the present review was to summarize the current knowledge of the carnosine’s biological properties in health and disease. Special attention is paid to the anti-inflammatory and antioxidant properties of carnosine, and its effect on muscle function as well as beneficial effects in various diseases. To obtain a comprehensive overview of the current data, the original research papers and clinical trials as well as systematic reviews available on the PubMed database were analyzed by using the following search terms: carnosine, functional food, muscle, health, disease, vascular, endothelium, inflammation, antioxidants, and oxidative stress. Only the English language literature that referred to both humans and experimental animals with no time restriction were reviewed. A total of 76 clinical trials were found using search terms carnosine and supplementation, most of them were related to carnosine muscle content and physical capacity/exercise in health. An additional 14 clinical trials and one systematic review with meta-analysis were identified in PubMed by search terms carnosine, aging, human. Limited number of clinical studies were related to diseases, mostly diabetes and/or obesity, as discussed further in the text.

## 2. Carnosine Metabolism

**Figure 1:**
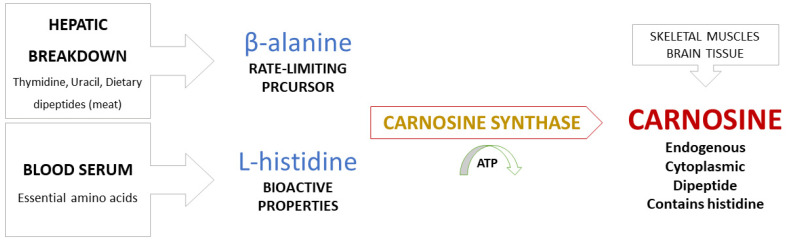
Synthesis of carnosine from precursor amino acids β-alanine and L-histidine.

*Metabolic pathways in synthesis of carnosine*. Carnosine is synthesized by bonding of the amino acids β-alanine (regulatory function) and L-histidine (biological activity), a reaction catalyzed by carnosine synthase [6,9,23]. β-alanine becomes available by hepatic breakdown of thymidine, uracil, and dietary dipeptides obtained from meat consumption and is considered to be a non-proteinogenic rate-limiting precursor of carnosine. L-histidine, on the other hand, is an essential amino acid present in serum and serves as a proteinogenic precursor with bioactive properties (Figure 1) [13].

**Figure 2:**
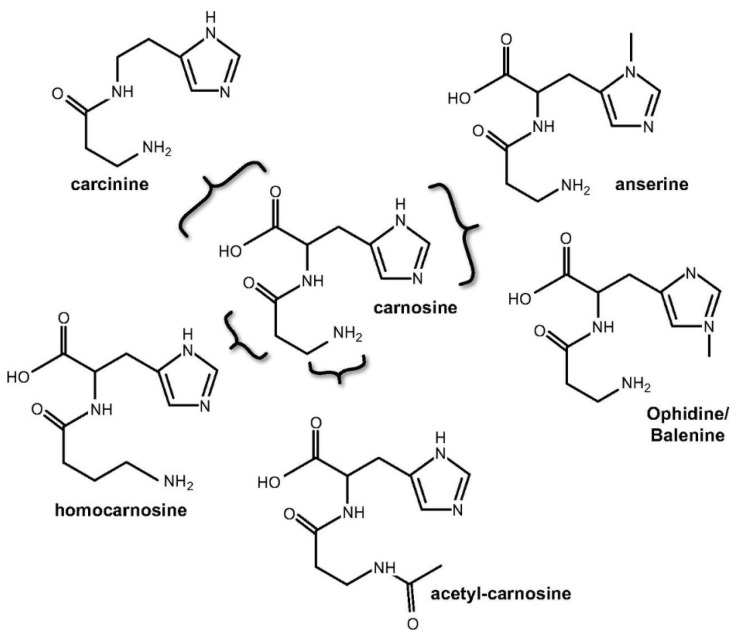
Chemical structures of carnosine and naturally occurring derivatives (figure adapted from Boldyrev et al. (2013) [3]).

Carnosine naturally occurs in numerous variants as carnosine derivatives including anserine and the ophidine/balenine-methylated imidazole ring of L-histidine; homocarnosine-GABA replaces β-alanine; carcinine–L-histidine is replaced by histamine; acetylcarnosine-acetylated β-alanine (Figure 2) [23]. Most common derivatives are mentioned methylated analogues, anserine, and ophidine, which are synthetized by carnosine *N*-methyltransferase mediated methylation of the imidazole ring. The same effect can also be achieved by enzymatic condensation of Npi-methylhistine with β-alanine through carnosine synthase, although the latter pathway is considered more physiologically relevant [3].

**Figure 3:**
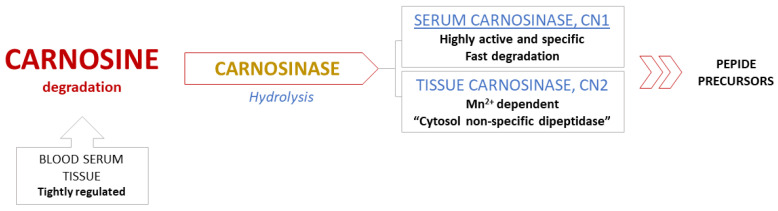
Degradation of carnosine.

*Metabolic pathways in breakdown of carnosine*. Degradation of carnosine occurs in serum and tissues through hydrolysis, catalyzed only by the enzyme carnosinase. Common peptidases have no effect on the breakdown of the carnosine molecule, while non-enzymatic degradation of carnosine is non-existent [3,6]. These specificities indicate an extremely well regulated metabolism, especially the degradation process (Figure 3) [13].

There are two human isoforms of enzyme carnosinase: serum carnosinase (CN1) and tissue carnosinase (CN2). Highly active and abundant CN1, which is found in serum and brain tissue, catalyzes degradation of both carnosine and homocarnosine, while Mn^2+^ dependent CN2 is expressed only in tissues and described as “cytosol nonspecific dipeptidase” [24]. High activity and selectivity of serum CN1 results in fast degradation of circulating carnosine in human blood within 2–3 h following a meal, which is the reason behind the inability to detect this dipeptide after the fasting period [13,25]. This feature certainly presents a challenge and substantially limits the possibility of future therapeutic applications of carnosine [26].

Furthermore, a recent study suggests that human kidneys possess their own internal system for carnosine metabolism since it was found that enzymes and histidine-containing dipeptides were detected in specific nephron compartments [27]. Since there was no correlation between carnosine concentrations and CN1 activity in kidneys as previously suspected, local tissue related impact probably has a crucial role in carnosine metabolism, specifically in diabetic nephropathy development [28].

## 3. Methodology of Carnosine Determination

One of the significant problems in clinical human studies is sampling and the method of determination of the compound of interest.

Carnosine sampling and sample preparation. The sampling can be divided in two main groups: tissue and liquid biopsy. The collected sample are commonly frozen in liquid nitrogen and stored at −85 °C until further preparation for analyses [29,30,31].

*Preparation of samples obtained by tissue biopsy.* The content of carnosine is mainly determined in the vastus lateralis muscle [32]. Harris et al. [32] acquired a biopsy sample from the vastus lateralis muscle of male subjects aged 20–35. Biopsy samples were taken by using the Bergström technique (Bergström muscle biopsy cannula). After gathering, the samples were stored in liquid nitrogen and freeze-dried. Single muscle fiber was weighed, extracted, and analyzed with HPLC, as described by Dunnett and Harris [33]. The extraction of carnosine from tissue samples is described in many papers and differs in a solvent and buffer. We are presenting a summary of different extraction methods in Table 1.

*Preparation of samples obtained by liquid biopsy.* Blood samples are usually collected for carnosine determination, but for certain experiments, carnosine is determined from cerebrospinal fluid (CSF) or urine [42]. Blancquaert et al. [37] collected samples of venous blood in EDTA-containing tubes, and samples were centrifuged. Deproteination of plasma was carried out using sulfosalicylic acid (35%), samples were centrifuged and the supernatant was used for further preparation and analysis [29,38,42]. Park et al. [18] determined carnosine concentration in human plasma after the dietary consumption of beef. Within one hour of sample collection, samples were centrifuged at 4 °C, and 5 mL of plasma was vortex-mixed with the addition of perchloric acid and heated in a boiling water bath. Samples were again centrifuged, and the supernatant was filtered and used for analysis.

Pandaya et al. [40] collected blood samples (in EDTA-containing tubes), plasma was separated and stored at −80 °C until further preparation for HPLC analysis. For analysis, 100 μL of plasma was taken. Proteins were precipitated by adding chilled methanol in a 1:4 ratio and separated by centrifuge. The collected supernatant was passed through the Costar spin column. The flow-through was speed-vac dried and reconstituted in 50 μL of water containing 3% ACN. LC-MS/MS was used for sample analysis.

### Analytical Methods for Carnosine Determination

*Nuclear magnetic resonance (NMR) spectroscopy* is a rapid method to provide complete structural analysis, with or without sample pre-treatment in both liquid and solid samples [43]. However, it is less sensitive than mass spectrometry.

Carnosine can be determined in tissues by non-invasive magnetic resonance imaging (MRI) and proton magnetic resonance spectroscopy (1H-MRS). In muscle, signal output predominantly originates from carnosine. However, in the brain, homocarnosine is predominant [44]. Özdemir et al. determined carnosine concentration in human muscle in vivo by proton magnetic resonance spectroscopy [45].

*HPLC* is the most commonly used method for determining carnosine, and it has an appropriate separation and selectivity accompanied with sufficient sensitivity provided by the large variety of detection methods. However, analysis of carnosine in complex samples by liquid chromatography can suffer stationary phase damage from matrix species and often requires the use of precolumn, large quantities of organic solvents, and bulky instrumentation [46]. A summary of different sample preparations and detections is presented in Table 2.

*Capillary electrophoresis and Microchip electrophoresis*. Nowadays, capillary electrophoresis (CE) and capillary electrochromatography (CEC) are recognized analogs of HPLC/UHPLC and nano-LC methods [47].

Carnosine can be determined in biological materials by capillary electrophoresis with UV detection [48], laser-induced fluorescence (LIF) mass spectrometry [49], and microchip electrophoresis (ME) with chemiluminescence [50] or capacitively coupled contactless conductivity detection (C4D) [51]. An important constraint of capillary electrophoresis is the limited sensitivity due to the small volume of the injected sample. Two different approaches can be employed for improving sensitivity: applying sensitive detectors such as laser-induced fluorescence and mass spectrometry or using preconcentration techniques such as sample stacking methods or isotachophoresis (ITP) [47]. Several techniques have been developed with improved sensitivity [48,50,52].

Microchip electrophoresis (ME) is a miniaturized mode of CE [53]. ME was first used to analyze carnosine by Zhao et al. in 2009 [50]. Chemiluminescent detection was used to determine the carnosine, anserine, and homocarnosine in biological materials [54]. For the carnosine to become detectable by the chemiluminescent detector, it needs to be labeled; for this purpose, *N*-(4-aminobutyl)-*N*-ethylsoluminol (ABEI) was used as a precolumn marker [54].

*MALDI-TOF-MS* is a new and powerful technique for examining biological samples and plays a significant role in modern life sciences [55]. Pisarev et al. [56] reported an ultra-sensitive method for the determination of carnosine. The limit of detection is in the picomolar range. Moreover, the concentration can be determined directly on site of a tissue sample without carnosine extraction. Even more sensitive methods have been developed by combining separation with electrospraying and detection by MS [57].

Electrochemical methods are rarely used for carnosine determination, but it is worth mentioning that they are widely used to characterize amino acids and peptides. Jozanović et al. [58] studied carnosine and anserine at the glassy carbon electrode with differential pulse voltammetry. They found that both dipeptides were oxidized, and their oxidation mechanism involves the transfer of one electron and one proton. The optimal pH for the oxidation of carnosine was 4.8 and for the oxidation of anserine it was 6.1, respectively. A linear relationship between the peak current and the carnosine and anserine concentration in the range of 1 × 10^−5^ M to 1 × 10^−4^ M was established.

## 4. Challenges in Carnosine Examination

Carnosine is a nutrient highly abundant in beef. Daily dietary intake of 30 g dried beef can completely supply daily carnosine requirement of the 70-kg adult to ameliorate human nutrition and health [59]. However, reported amounts of carnosine in beef meat are very inconsistent in the literature and may vary greatly [60] from 14 mg [61] to 1 g [62] of carnosine in 100 g wet beef meat. It is assumed that such large differences in nutrient composition may result, in part, from analytical methods or beef breeds [59]. Interestingly, it is suggested that some components in beef inhibit serum carnosinase [3], causing an elevated circulating amount of carnosine that may directly endue the dipeptide to extraintestinal tissues and cells. On the other hand, it is difficult to estimate the extent to which substances from food (anserine, amino acids (e.g., histidine and β-alanine), and copper) affect carnosinase activity. Furthermore, a particular challenge is to determine the basal amount of carnosine in the human body, since carnosine is negligible or not detectable in the human plasma, and muscle biopsy is an invasive procedure.

The next important challenge lies in the fact that β-alanine availability primarily limits carnosine synthesis in human skeletal muscle, but a diet sufficient supply of histidine is also important for carnosine synthesis [34,63]. Studies reported that in humans consuming enough histidine from animal-source foods that histidine is not a limiting factor in the synthesis of carnosine, and in nonvegetarian adults, an equal intake of β-alanine and carnosine successfully enhances muscular carnosine amount to the identic extent. However, in vegetarians, it is unrevealed whether histidine dietary intake limits carnosine synthesis with or without β-alanine supplementation [64].

## 5. Carnosine Biological Effects

### 5.1. Carnosine in Muscle Function

Although it is well known that carnosine plays an important role in exercise performance and skeletal muscle homeostasis [65], its definitive role is still not established [3]. Carnosine functions as a high-energy phosphate system comparable to the creatine/phosphocreatine system [66]. In their experiments, Severin et al. [67] demonstrated that carnosine plays a role in contractile function of skeletal muscle. In nerve-muscle preparation of frogs, it has been shown that the offset of the fatigue could occur during rhythmic nerve-stimulated muscle contractions if 10 mM of carnosine was added to the surrounding medium. This is also true for anserine [68]. Below, we summarize the currently best-established physiological roles of carnosine in skeletal muscle.

*Intramyocellular pH buffering*. The role of carnosine as a pH buffer was first proposed in the early works of Bate Smith [69] and Deutcsh and Eggleton [70]. Although the specific mechanism is still unknown, it has been established that increasing carnosine via β-alanine supplementation improves exercise capacity and performance in exercise in durations of 30 s to 10 min [71]. This result is consistent with carnosine acting as a pH buffer because H^+^ concentrations in muscle are highest after exercise. The greatest benefit for exercise capacity is shown from increasing carnosine content [72]. Carnosine is a mobile buffer that can dissolve in cytoplasm, whereas proteins are fixed buffers [73]. Carnosine contribution to pH homeostasis is allowed by such mobility [74]. When the amount of carnosine in skeletal muscle is increased by nutritional intervention, the level of blood acidosis during high-intensity exercise is reduced in humans [75].

In addition to its buffering effects in muscle, carnosine protects against reactive oxygen species (ROS) produced during an exercise as reported in Dawson et al. [76]. Their study on downhill running in rats showed that a supplementation-induced increase in muscle histidine-containing dipeptides could diminish the production of thiobarbituric acid reactive substances (TBARS, lipid peroxidation products). This observation is consistent with earlier studies reporting a protecting function of carnosine on lipid peroxidation in working muscles [77].

*Effect on Ca^2+^ handling and muscle contractility*. Regulation of the excitation–contraction coupling in skeletal muscle is a complex sequence of events in which calcium is released from the sarcoplasmic reticulum (SR) through ryanodine receptors (RyR). Calcium binding to troponin C allows cross-bridge formation and production of force, and after that, the reuptake of calcium in the SR terminates contraction [78]. Carnosine may be included in the regulation of several of these steps. Release of Ca^2+^ induced by carnosine in SR vesicles from rabbit white skeletal muscles was demonstrated by Batrukova and Rubtsov [79] with constituent amino acids L-histidine and β-alanine. Similar evidence was presented in a study on chemically skinned fibers of human vastus lateralis muscle [80]. Considering that Mg^2+^ has a strong inhibitory effect on RyR [81], these findings were challenged [82], arguing that Mg^2+^ levels were abnormally low in previous studies and they demonstrated that in physiological levels of Mg^2+^, carnosine does not play a role in stimulating the RyRs. Calcium sensitivity of contractile apparatus is probably even more important for carnosine’s effect on excitation–contraction coupling. These were demonstrated in studies on animals, but also in studies on skinned human muscle fibers [83,84].

*Cytoplasmic Ca^2+^-H^+^ exchanger.* Carnosine may function as a diffusible cytoplasmic Ca^2+^–H exchanger in cardiomyocytes [74]. Elements of two previously discussed roles are combined in this function: Ca^2+^ and pH buffering. The interrelationship between H^+^ and Ca^2+^ has an important role in exercising skeletal muscle because H^+^ can compete with Ca^2+^ at the troponin-binding site and by that, limit the ability of the muscle contractile machinery to operate effectively [85]. Ability of Ca^2+^ and H^+^ to competitively bind to carnosine can cause unloading of Ca^2+^ in areas of high H^+^ production and the other way around [74]. A role of carnosine as the Ca^2+^–H^+^ exchanger in human skeletal muscle could be explained by the fact that an increase in carnosine level can reduce half-relaxation time [86]. The main role in relaxation time has the rate of dissociation of Ca^2+^ from troponin, translocation of Ca^2+^ to a site close to the SR, and reuptake of Ca^2+^ into the SR by SERCA pumps (SR Ca^2+^-ATPase) [82]. Carnosine, as a mobile buffer, may translocate Ca^2+^ closer to the SERCA pump for Ca^2+^ reuptake. This was demonstrated in rat ventricular cardiomyocytes [74], where spatiotemporal responses in Ca^2+^ stimuli are different from skeletal muscle [68,87]. It is possible that this also occurs in human skeletal muscle, but validation is required before making strong conclusions.

*Muscle as storage and release organ for carnosine.* Muscle contains most of the carnosine present in the organism, and it is tempting to speculate on the possible role of skeletal muscle as a depot and donor of carnosine. Carnosine level in human skeletal muscle (without carnosine or β-alanine supplementation) is in the range from 5 to 10 mM [3], or 16.7–33.3 mmol/kg dry weight of muscle. Carnosine amount depends on muscular activity and bodybuilders may have average values from 13 mmol or 43 mmol/kg dry weight [88]. One of the early discoveries were that tissues that are capable of synthesizing carnosine should not be able to provide for its degradation [68]. Carnosine could play an autocrine, paracrine, or even endocrine role, especially during exercise. This was supported by the findings of Nagai et al. [89] who detected a doubled carnosine concentration in plasma of rats who were trained on a running wheel compared with sedentary rats. It was also shown that in human skeletal muscle during knee extension exercise at 20 W, carnosine content markedly increases in the interstitial fluid, determined by microdialysis [90]. It is still unknown whether carnosine exempt from muscle cells is an outcome of a functional transporter acting or a result of sarcolemmal rupture [3]. This contraction-included release system is yet inaccessible, but may be linked to the interaction between carnosine, histidine, and histamine [91].

*Direct effects on aerobic metabolism and glycolysis.* Early research showed that in isolated skeletal muscle, carnosine regulates enzyme activity and chelates heavy metal glycolytic inhibitors, which leads to an increase in glycolytic flux [92,93]. Despite success in vitro, data of direct influence on energy metabolism in human skeletal muscle are equivocal. Independently of oxidative capacity, increase in glycolytic flux or capacity is quantifiable by higher lactate accumulation during and after exercise. Some of the studies with β-alanine supplementation has shown this with an increase in post exercise plasma lactate values. Some studies measured blood lactate after a special judo fitness test [94] and 4 × 30 s upper-body Wingate test [95]. However, in these studies, direct effects on glycolysis could not be separated from indirect effects because the total mechanical work was not matched between pre-supplementation and post-supplementation trials. Only a few studies have quantified the effects of β-alanine supplementation on energy system contribution with matched total mechanical work and reported that after a high-intensity intermittent cycling test, a decrease in post exercise muscle lactate and oxygen deficit was detected [96]. Additionally, a small (1.3%) but significant increase in the estimated aerobic energy contribution was observed. Another study using treadmill running showed that β-alanine supplementation induced delay in the onset of blood lactate accumulation and indicated an improvement in oxidative capacity [97]. Despite good results in vitro, the influence of carnosine as a direct modulator of energy metabolism in skeletal muscle appears to be less noticeable.

Carnosine content in skeletal muscle depends on a variety of individual factors including age, gender, diet, distribution, muscle fiber distribution, and training status. The carnosine content in type II fibers is approximately 30–100% higher than the carnosine content in type I fibers, further demonstrating the importance of carnosine during high-intensity activity [34,35,98].

β-alanine or carnosine dietary supplementation improves muscular performance in humans [65]. It is reported that β-alanine dietary supplementation (6 g/day for 23 days) decreases the histidine plasma and muscle concentrations [37], whereas oral administration of carnosine enhances the β-alanine but also histidine levels in the human plasma [99], suggesting the advantage of the carnosine supplementation (synthetic or carnosine-rich foods) over the consumption of β-alanine alone.

Definitive role of carnosine in skeletal muscle is still unknown, but earlier described functions are possibly the most important roles of carnosine in skeletal muscle. Other potential roles of carnosine are under investigation such as activation of myosin ATPase, effects on glycolysis, neuromuscular junction, and mitochondrial respiration [3]. Those potential roles of carnosine are up to this day still poorly understood.

### 5.2. Antioxidant and Anti-Inflammatory Properties of Carnosine

**Figure 4:**
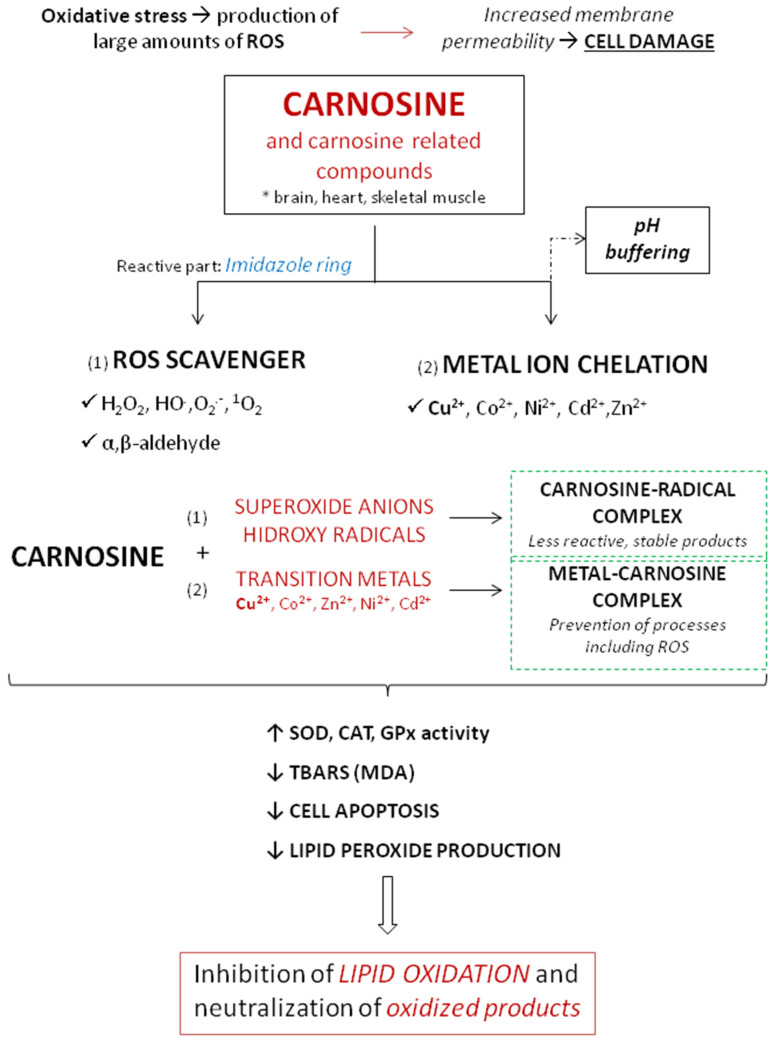
Schematic overview of the antioxidant effects of carnosine.

The antioxidant activity of carnosine is mediated by different mechanisms involving metal ion chelation, and scavenging of ROS and peroxyl radicals [3]. The most important antioxidant effects of carnosine are summarized in Figure 4.

A study that examined the effect of carnosine on oxidative stress in human kidney tubular epithelial (HK2) cells indicated that carnosine decreased NADPH oxidase (Nox) 4 expression and increased total superoxide dismutase (T-SOD) activity, thus reducing the production of intracellular ROS, relieving the oxidative stress of cells, and ultimately inhibiting the mitochondrial pathway of apoptosis [100].

Ability of carnosine to protect against pathologies characterized by oxidative stress has been shown in a number of conditions. Products of such modification usually lose ability to carry out their functions [101]. Carnosine changes the reactivity of superoxide anion by forming a charge–transfer complex with the superoxide radical [102] and also by reducing the efficiency of hydroxyl radicals, creating a compound less reactive than the hydroxyl radical [103]. One of the mechanisms to protect organisms from oxidative stress is the chelation of transition metals, preventing them from participating in deleterious processes involving ROS [104,105]. In the case of the Cu^2+^–carnosine complex, Cu^2+^ is bound to some organic compounds like imidazole, forming two six-membered chelate rings [106]. In the presence of copper, the fast oxidation of carnosine by ·OH still takes place and yields adducts of ·OH radicals at the imidazole ring. Imidazole compounds are known to react with singlet oxygen forming endoperoxide products and that group is the preferred site for ·OH binding in the presence of copper [107]. Regarding this, carnosine showed an efficient chelator of copper and other transition metals and reacted with singlet oxygen several times faster than other tested parameters (like L-histidine) [108]. Interestingly, when comparing metals involved in free radical generation, carnosine was found to have a greater antioxidant activity coupled with copper than iron [109]. Furthermore, Decker et al. showed that purified carnosine scavenges peroxyl radicals and that scavenging is largely due to the L-histidine residue. Carnosine does not act as an electrotransfer agent because they were not able to reduce MbFe(III) to MbFe(II)O_2_, as shown in the model of Zucker obese rats [110].

At physiological concentrations, carnosine directly reacts with superoxide anion similar to ascorbic acid [111]. In physiological conditions, carnosine was found to reduce oxidative damage and to improve antioxidant activity of different antioxidative enzymes (such as SOD, GPx…) [11]. Experiments on aged rats showed that therapy with 250 mg/kg/carnosine per day significantly decreased oxidative stress and increased activity of antioxidative enzymes [112]. The same effect was not found in young rats. In similar model of aged rats, carnosine increased liver vitamin E, which further demonstrates its importance in defending the organism from free radicals [112].

Rising data indicate that carnosine acts as a scavenger of reactive and cytotoxic carbonyl species including 4-hydroxynonenal (HNE) [113]. HNE is an aldehyde generated endogenously by lipid peroxidation of unsaturated fatty acids [114] that act as ‘toxic second messengers’, extending the harmful potential of free radicals [115]. HNE is considered an important biomarker of oxidative stress and accumulating data indicate that it may modulate signaling pathways of cell proliferation, apoptosis, and inflammation [116]. At low and physiological levels, HNE acts as an endogenous signaling molecule, but at high concentrations, HNE is involved in the onset of several chronic diseases [117]. Because biological activity of HNE is related to its intracellular levels, the processes of maintaining its intracellular concentrations are most important, both in the defense against oxidative stress but also in the pathophysiology of several disease processes [117]. Carnosine can form conjugates with HNE and, therefore could prevent the toxicity of HNE and associated aldehydes [118]. Carnosine adduction to HNE appears to start with the generation of a reversible α,β-unsaturated imine, followed by ring closure through an intra-molecular Michael addition [113]. In therapeutic observation, it was demonstrated that dietary carnosine supplementation prevents atherogenesis by facilitating aldehyde removal from atherosclerotic lesions in apolipoprotein E-null mice [119]. Taken together, carnosine endogenous concentration may be significant determinants in the formation of atherosclerotic lesion, and carnosine treatment could be a valuable approach for the prevention or treatment of atherosclerosis [119].

Furthermore, the accumulation of HNE has been extensively documented in blood and tissue samples from obese/diabetic patients [120,121], and emerging studies have indicated that these reactive aldehydes are more than simply markers of oxidative stress. Rather, it is suggested that these reactive species may play a significant pathogenic role in obesity-associated disorders such as insulin resistance [122,123], and a carnosine analog alleviates the production or enhances the removal of reactive carbonyl species, providing promising new therapeutic compounds for cardiovascular and metabolic diseases related to obesity [123].

Through its effect on the immune system, carnosine decreases both oxidative stress and inflammation, as demonstrated in the model of amyloid-induced inflammation [124]. The mechanism of this carnosine action is the interaction with specific receptors localized on the cell membrane, which modulate macrophage function by increasing their phagocytotic activity [125]. In addition, a study on the RAW 264.7 (murine macrophage cells) cell model showed that carnosine reduced phorbol 12-myristate 13-acetate (PMA) induced oxidative stress in macrophages by decreasing the expression of Nox1 and Nox2 genes as well as the intracellular superoxide anion levels [124].

### 5.3. Immunomodulation

Numerous studies provide evidence of the beneficial effect of carnosine supplementation on cytokine release, and consequently inflammation in humans and animals as follows [126,127,128,129,130]. For example, carnosine can lower renal interleukin-6 (IL-6) and tumor necrosis factor-alpha (TNF-α) in rats after nickel-induced nephrotoxicity [131].

Elevated levels of pro-inflammatory cytokines such as IL-6 and TNF-α are usually related to insulin resistance and alteration of insulin sensitivity in diabetic patients, while they can also be used as markers of renal damage in relation to diabetic nephropathy [131,132,133,134]. Hence, the in vivo effect of carnosine and histidine supplementation through drinking water was studied in diabetic Balb/cA mice [135], and these studies suggest that increased IL-6 and TNF-α in diabetic mice were significantly suppressed by the intake of histidine or carnosine.

Several human pilot studies have investigated the effect of carnosine supplementation [59,136,137,138]. An increase in fasting insulin levels and insulin resistance was hampered in individuals receiving carnosine supplementation [136].

Additionally, it has been suggested that carnosine supplementation prevented worsening of insulin resistance in non-diabetic overweight and obese individuals [136], but also carnosine may have beneficial effects on the plasma lipidome [137]. Since excessive generation of IL-6 and TNF-α compromise insulin sensitivity, suppression of IL-6 and TNF-α by carnosine and histidine can have beneficial effects on reducing or preventing diabetes-related complications as well as preventing altered endothelial function by inflammation.

Overexpression of pro-inflammatory IL-8, along ide IL-6 and TNF-α, has been associated with inflammatory processes in the digestive system, particularly in the intestines, inducing inflammatory bowel disease (IBD) [139]. Several studies have found that carnosine inhibits hydrogen peroxide and TNF-α stimulated secretion alongside mRNA expression of IL-8 in human intestinal epithelial cells [140,141]. In ulcerative colitis, as one of the inflammatory bowel diseases, research is being conducted to predict disease relapse. Reduced plasma histidine levels, which is an integral part of carnosine, predicts the risk of ulcerative collitis relapse [142].

In a similar manner, carnosine and histidine supplementation exhibited a hepatoprotective effect. Specifically, results of studies conducted on male Balb/cA mice with ethanol- or acetaminophen-induced chronic liver injury indicated that both pre-(acetaminophen) and post-intake (ethanol) of carnosine and histidine decreased hepatic levels of IL-6 and TNF-α [143,144].

Potential beneficial impacts of carnosine and derivative anserine on inflammatory cytokine secretion was observed in neurodegenerative disorders, specifically Alzheimer’s disease (AD) and Parkinson’s disease (PD) [127,145]. The mentioned animal study was carried out on C57BL/6 mice with PD induced by administration of 1-methyl-4-phenyl-1,2,3,6-tetrahydropyridine (MPTP), while a human study examined the effect on preserving cognitive function in elderly volunteers. It was found that anserine and/or carnosine supplementation significantly decreased IL-6, TNF-α, and IL-1β in pre-treated mice with MPTP-induced PD, while in human subjects, there was a significant decrease of IL-8 and IL-5 detected in the treated group [72,146].

Animal studies have shown that carnosine has a beneficial effect on reducing acute kidney injury due to septic shock [147,148]. Carnosine renormalized lipid peroxidation, reduced the level of IL-β, tumor necrosis factor-alpha (TNF-α), and macrophage inhibitory factor (MIF), thus demonstrating a beneficial effect in sepsis [149].

Although there are studies that suggest a potential beneficial role of carnosine in clinical conditions, there is insufficient data and there are still many unknowns. Additional experimental and clinical research in humans is needed in order to elucidate the mechanisms of carnosine application in these diseases as well as to more stringently test for clinical efficacy and potential adverse effects.

### 5.4. Carnosine and Aging

There are very few clinical trials concerned with the effects of β-alanine or carnosine supplementation on exercise capacity or the muscle carnosine content in elderly subjects. A PubMed database search with terms carnosine, aging, human, restricted to clinical trials, retrieved 14 publications. One trial was excluded due to the age of the participants (mean 25 years) and five others due to topic (skin health or vision) and another one was excluded due to the inability to obtain access to the full text and the paper was a combination of a review and pilot study. In the other analyzed clinical trials, the supplementation protocols differed, mainly including at least a month of beta-alanine or carnosine intake from 800 mg to 2.5 g/day. It has been demonstrated that β-alanine supplementation increased exercise capacity and improved the ability of β-alanine to extend exercise duration [150]. Twenty-eight days of 800 mg/day of β-alanine supplementation increased cycling performance via an enhanced time to exhaustion and total work completed with associated lactate clearance during passive rest in female master athletes [151] and increased their peak torque and work completed, indicating β-alanine improves lower-body exercise performance [152]. Another study in eighteen healthy elderly subjects of both sexes showed that β-alanine intake was effective in increasing the muscle carnosine content, subsequently improving their exercise capacity [153]. Taken together, these results are promising, suggesting that supplementation of carnosine or its precursors could have a beneficial effect on muscle strength, endurance, and improved functionality in elderly people. However, due to a small number of involved subjects and diversity in their locomotor capacity at the enrollment, it is necessary to conduct research on a much bigger scale. In addition, there has been no study investigating the effects of consuming functional food enriched with carnosine or beta-alanine on physical performance in an elderly population.

Another important topic in aging is preservation of cognitive function and the potential of carnosine as an antioxidant to contribute to this goal. Sixty healthy elderly volunteers participated in a double-blind randomized controlled trial, receiving 1.0 g of anserine/carnosine (ASC) (3:1) for three months. There was a significant correlation between the preservation of verbal memory and suppression of CCL24 expression in persons taking ACS, suggesting that ACS intake preserves verbal episodic memory, probably due to inflammatory chemokine -CCL24 suppression in the blood [130], in agreement with a previous finding that ACS treatment decreases the production of CCL-2, IL-8 in elderly people and improves brain perfusion [145]. Nutraceutical supplements containing carnosine have been shown to improve some cognitive functional tests in the elderly [154], while recent systematic review and meta-analysis suggest that administration of carnosine/anserine for 12 weeks at a dose of 1 g/day may improve global cognitive function, but not depressive symptoms in elderly subjects [155].

## 6. Carnosine Supplementation

*Chicken meat supplemented with carnosine.* Supplementation with carnosine (and other histidine-containing dipeptides as well as their components) have been proposed as potential strategies for the prevention of chronic diseases [156]. Native chicken is an important possible source of functional food because it contains considerable amounts of bioactive compounds when compared with commercial broiler chicken and other meat [157]. Due to the above demonstrated beneficial effects of carnosine on metabolism in many organs and tissue, there has been an attempt to produce meat enriched with carnosine (so called functional food). For example, Kai et al. [158] investigated the influence of the three levels of histidine in broiler feed: 67% (low), 100% (control), and 200% (high) compared to NRC recommendations (1994). They found out that carnosine and anserine levels in muscle control groups of chickens were 1434 ± 86.3 and 5902 ± 153.5 µg/g of muscle, while high levels of histidine in feed increased the content of carnosine to 2464, 8 ± 185.6, and anserine 6652.5 ± 287.8 µg/g of muscle. However, Tomonaga et al. [159] found that histidine supplementation in chicken feed increased only carnosine content, but not anserine content. The muscles of the breast contain higher concentrations of carnosine compared to the muscles of the drumstick and thighs [160]. Some authors such as Kralik et al. [161] increased the β-alanine (0.0; 0.5 and 1.0%) and L-histidine (0.0; 0.3 and 0.5%) concentration in feed aiming to increase the content of carnosine (Table 3). They found a more efficient effect of higher amino acid concentrations on carnosine deposition in the chicken breast muscles. The carnosine concentration in the poultry body can be modified by adding amino acids (β-alanine and L-histidine) individually or together to the feed [3,162]. According to Harris et al. [163], the rate of carnosine synthesis in muscle is more affected by β-alanine than l-histidine. Chicken meat is liable to oxidative processes that can adversely affect color, taste, and odor. Due to its antioxidant activity, carnosine is important in prolonged meat storage [164]. The importance of β-alanine in carnosine synthesis was reported by Qi et al. [165] and Perin et al. [166]. Chicken meat quality can be improved by antioxidants in feed since, after digestion, they are incorporated into cell membranes and thus protect the tissue from oxidation (i.e., from reactive oxygen species). Carnosine, being a natural dipeptide, is effective in reducing lipid oxidation and maintaining meat quality. Qi et al. [165] found that the addition of 0.5% carnosine in the chicken feed decreased TBARS (thiobarbituric acid reactive substances) and increased TAOC (total antioxidant capacity) in blood and chicken muscles (*p* < 0.001), improving the meat quality. Similarities were observed in studies by Cong et al. [167] who established that the thigh muscles contained carnosine of 1509, 1539, 1569, and 1617 mg/kg and increased concentrations of carnosine synthase 1.0, 1.08, 1.15, and 1.22 mg/kg. Kralik et al. [168] investigated the impact of amino acid and MgO addition on carnosine deposition in chicken meat (Table 3).

The authors found an influence of feeding treatments on the technological properties of meat, the carnosine concentration in the breast and thigh muscles as well as the level of TBARS values in fresh and frozen meat [168]. These results are in line with those reported by Soyer et al. [169] and Cong et al. [167,170]. Analysis of carnosine content results in breast muscle tissue showed that carnosine content increased compared to the P1 control group.

## 7. Forthcoming Research—Potential Health Benefits and Clinical Use

### 7.1. Carnosine in Glucose Metabolism/Diabetes

Type 2 diabetes mellitus is one of the leading public health problems and a global epidemic in modern society. Despite all methods of treatment, which are often insufficient, diabetes is associated with an increased risk of cardiovascular diseases, renal failure, visual impairment, and limb amputations [171]. Known as a natural antioxidant, carnosine has been found to improve metabolic control in different animal models of diabetes, despite the fact that its mechanisms are still not completely known. There are several studies about the effect of carnosine supplementation in animals with metabolic syndrome or diabetes (Table 4). Carnosine in animal models demonstrated protective effects on the development of chronic complications in diabetes [89,172,173,174]. Treatment of rats with a high-fat high-carbohydrate diet was associated with the development of metabolic syndrome, and carnosine supplementation reduced abdominal obesity, blood pressure values, glucose levels, and normalized total cholesterol and low-density lipoprotein (LDL) levels [175]. Furthermore, carnosine supplementation showed positive effects in mice with diabetic nephropathy by restoring the glomerular ultrastructure, which reduced albuminuria [176]. It was observed that carnosine treatment decreased proteinuria and renal damage in diabetic mice [177], inhibited the production of fibronectin and TGF β in renal cells [178], and also improved wound healing in diabetes [20]. Similarly, in rat models of type 2 diabetes mellitus, carnosine supplementation decreased serum lipids, creatinine, and urea levels [179].

Carnosine has been demonstrated to decrease the circulating Insulin Like Growth Factor Binding Protein 1 (IGFBP1) levels and the liver expression of IGFBP1, possibly directly by suppressing HIF-1a-mediated IGFBP1 induction and in an indirect manner by increasing circulating insulin level followed by a decrease in the blood glucose levels and increased plasma levels or IGF1. Reduction of IGFBP1 in diabetes through both insulin-dependent and insulin-independent pathways is a novel mechanism by which carnosine contributes to the improvement of the metabolic control in diabetes [180]. Several human studies involving patients with type 2 diabetes mellitus have also shown positive effects of carnosine supplementation (Table 5). In a randomized double-blind study by Karkabounas et al. [181], obese type 2 diabetic patients were either supplemented daily with α-lipoic acid, carnosine, and thiamine or placebo. Results showed reduced glucose and HbA1c levels after supplementation, probably by increasing insulin production from pancreas. In another randomized, double-blind, placebo-controlled clinical trial, patients with type 2 diabetes mellitus received either placebo or carnosine. Patients who received carnosine had a significant decrease in fat mass, fasting blood glucose, glycated hemoglobin, and serum levels of triglycerides [129]. On the other hand, carnosine supplementation resulted in a decrease in HbA1c, but had no effect on cholesterol, fasting blood sugar, triglycerides, and high density lipoprotein –C (HDL-C) [182]. These studies are promising, but more research is needed on larger samples. As the number of patients with diabetes and metabolic syndrome is increasing, this is an interesting area for future research.

### 7.2. Carnosine in Cardiovascular Disorders (Atherosclerosis, Heart Failure)

Cardiovascular disease remains the leading cause of death globally [183]. There are many factors that promote the development of endothelial dysfunction underlying cardiovascular diseases such as hypercholesterolemia, diabetes mellitus, metabolic syndrome, hypertension, aging (advanced glycation end products), oxidative stress, proinflammatory cytokines, etc. [184]. Due to its anti-inflammatory, antioxidant, anti-glycation, anti-ischemic, and chelating roles in body, carnosine may be useful in treating cardiovascular diseases [179]. Macrophages have a pivotal function in various diseases associated with oxidative stress and inflammation (atherosclerosis, diabetes) [185], and it is reported that carnosine has beneficial effects on macrophage cells under oxidative stress and inflammation conditions [186]. Carnosine was able to block the formation of catecholaldehyde protein adducts (which have been implicated as causal factors in the etiology of neurodegenerative diseases and cardiac injury from ischemia and diabetes) in isolated human cardiac mitochondria treated with norepinephrine, which can be a therapeutic potential of carnosine in diseases associated with catecholamine-related toxicity [187]. In patients with heart failure who were treated with carnosine for six months, an improvement was seen in the 6-min walk test distance and quality of life by the EQ-5D questionnaire and visual analog scale score (VAS). Furthermore, peak VO_2_ and peak exercise workload also increased in the treatment group [188].

The studies shown in Table 6 were performed on a small sample and mainly on animal models or in vitro, so it is necessary to further investigate the effect of carnosine in randomized controlled clinical trials.

### 7.3. Carnosine in Neurological Disorders

According to previous research, overproduction of reactive oxygen (ROS) and nitrogen species (RNS) and failure of balancing the effects of endogenous antioxidant defenses contribute to the oxidation of mitochondrial proteins, lipids, deoxyribonukleic/ribonucleic acid, and thus the development of neurodegenerative diseases [190,191]. Today, the effectiveness of carnosine on various neurological and mental disorders (like Alzheimer’s diseases, acute ischemic stroke, schizophrenia, depression) is being increasingly examined [192,193,194,195]. Carnosine has been shown to have positive effects, strengthening the endogenous antioxidant protection of the organism [196], increased activity of superoxide dismutase in erythrocytes [197], decrease in hydroperoxides in lipoproteins of blood plasma, considerably increased the resistance low-density and very-low-density lipoproteins against Fe^2+^-induced oxidation as well as a reduction in the amount of oxidized proteins in blood plasma [197]. Altogether, carnosine, through its reduction of oxidative stress, decreases neurologic symptoms, negative consequences of therapy, and improves cognitive functions of the brain in patients [192,193,194].

The mechanisms of carnosine-induced neuroprotection in brain ischemia have been described in several animal studies. According to the results of a meta-analysis of eight animal studies exploring the effects of carnosine on ischemic stroke, there was a 29.4% average reduction in infarct volume with a clear dose-dependent effect (38.1% reduction on 1000 mg/kg dose compared with 13.2% for doses less than 500 mg/kg) [198]. In order to clarify the effects of carnosine on brain ischemia, several mechanisms have been proposed. A pre-treatment comprising a formula NT-020 (a pill-based nutraceutical containing a proprietary formulation of blueberry, carnosine, green tea, vitamin D3 and Biovin) for a duration of two weeks before transient occlusion of the middle cerebral artery in rats resulted in the reduction of motor asymmetry and neurological dysfunction, decreasing the striatum ischemic area. In comparison to placebo supplemented rats, this was accompanied with a respective one-fold and three-fold increase in neurogenesis in the subventricular zone and ischemic striatal penumbra, which might be ascribed to growth factors including glial cell-derived neurotrophic factor, the stem cell factor (SCF), and the vascular endothelial growth factor (VEGF) [199]. There is another mechanism related to the anti-inflammatory properties of carnosine. In a permanent cerebral ischemia model, the dose-dependent supplementation with a beef decoction (with 63% of its amino acid content being carnosine) resulted in the downregulation of the expression of proinflammatory cytokines IL-6, TNF-α, and interferon-γ (IFN-γ) as well as the upregulation of the expression of anti-inflammatory cytokine IL-4 [200]. It was also demonstrated that the carnosine treatment before brain ischemia reduced lipid peroxidation and enhanced antioxidant activity in the hippocampus and prefrontal cortex, inhibited mRNA expression of mediators of apoptosis (apoptosis-inducing factor, AIF, caspase 3), and upregulated mRNA expression of STAT3, which is an important regulator of anti-apoptotic factors [80,201,202]. On the other hand, anserine (β-alanyl-3-methyl-L-histidine) and N-acetyl carnosine, which are carnosine analogues, did not reduce infarct volume or improve neurologic function in a mouse model of focal cerebral ischemia [203].

Some animal studies propose that the brain histaminergic system is connected with epilepsy [204,205]. After being supplemented for 45 days with a compound comprising homocarnosine, carnosine, and anserine, the animals displayed significantly increased anti-convulsant activity after the epilepsy was induced using pentylenenetrazol (PTZ) as well as reduced lipid peroxidation and increased antioxidant concentrations [206]. Carnosine dose-dependently reduced seizure severity and prolonged the latency for myoclonic jerks after the beginning of seizures. Carnosine also increased the levels of histamine in the hippocampus and cortex that were decreased after administration of PTZ [207,208,209,210]. Furthermore, carnosine precursor L-histidine was a beneficial adjuvant to the anti-epileptic drugs carbamazepime and phenytoin in the case of mouse models of maximal electroshock-induced seizures [211]. In addition to the carnosine-histidine-histamine pathway, carnosine can also have a direct impact on CA1 pyramidal neurons [212] or act as a precursor for the neurotransmitter GABA [213] and in that manner, mediates its anti-convulsant properties.

Alzheimer’s disease (AD) is a neurodegenerative disease characterized by loss of cognitive abilities [214]. An increasing body of evidence shows that supplementation with carnosine, anserine, or its precursors could impede AD pathogenesis at the neurotoxic protein formation level. For example, mice with AD and mice with knockout of beta secretase 1, a key enzyme in the clearance of β-amyloid, exhibit reduced carnosine brain content [215]. In addition, β-alanine levels may be important in the modulation of spatial memory [216]. The addition of anserine, a methylated form of carnosine to AD-model mice for eight weeks ameliorated pericyte coverage on endothelial cells, reduced chronic neuroinflammation of glial cells, and thus improved their memory function [217]. Several studies have reported that carnosine supplementation reduced β-amyloid cumulation in the hippocampus of a transgenic mouse model of AD [218]. The finding that metabolic disturbance plays an important role in AD pathogenesis is supported by the discovery that advanced glycation end-products (AGEs) found in β-amyloid plaques [219] and pre-incubation of β-amyloid with carbohydrates promoted transformation of soluble β-amyloid into insoluble deposits, accelerating the β-amyloid aggregation [220]. Several studies have proposed that the carnosine mechanisms in neurodegeneration and in metabolism might overlap. These mechanisms encompass decreasing oxidative stress with concomitant strengthening of the antioxidant system and anti-inflammatory response or enhanced activity of acetylcholinesterase [221,222,223]. Carnosine has a protective effect on BV-2 microglia cells by reducing the levels of reactive NO and O_2_ species, and the expression of inducible nitric oxide synthase (iNOS) and NADPH oxidase. Furthermore, it reduces the secretion of pro-inflammatory cytokines such as IL-1β, and increases the release of transforming growth factor-beta 1 (TGF-β1), and through these mechanisms, prevents neuronal dysfunction in the brain of a person with AD [124]. Interestingly, Hata et al. [224] examined the association between serum concentrations of β-alanine, carnosine, and anserine metabolites, and the risks of dementia in the general elderly Japanese population and concluded that higher carnosine/anserine intake may be beneficial for dementia prevention. Taking into account that BA/carnosine supplementation improves cognitive function in elderly people, one may hypothesize that carnosine intake may also have an important beneficiary effect in people with AD or dementia of other causes. Only four clinical trials were identified in PubMed that evaluated the effects of carnosine supplementation in dementia. In the group of mild cognitive impaired (MCI) people, anserine/carnosine intake improved the score in the global Clinical Dementia Rating compared to the placebo taking group, which was even more prominent within the subgroup of APOE4(+) subjects with MCI [225]. Anserine has also been shown to protect elderly persons with MCI from cognitive decline. Protection occurs due to suppression of myeloperoxidase-mediated neuroinflammatory responses due to the scavenging properties of anserine toward hypochlorous acid [226]. On the other hand, treatment with a formula containing carnosine did not exhibit a significant effect in a population of patients with moderate AD [227]. No data are available on the carnosine intake and AD or dementia course/outcome in a human population and there is a lack of any randomized controlled trials that would confirm the beneficiary effects of carnosine and its precursors on cognitive function or the mechanisms of neuronal function, observed in animal models.

### 7.4. Carnosine in Malignant Diseases

The positive effects of carnosine in the treatment of tumors are increasingly being observed. It is likely that L-carnosine inhibits splenic sympathetic nerve activity and proliferation in rats as well as the multiplication and growth of cancer cells by increasing the natural killer cells (NK cells) responsible for the natural cytotoxicity of the organism [30]. Carnosine combined with radiotherapy reduced the side effects of radiation, injury of skin and intoxication of the organism, increased immunity, decreased the level of reactive oxygen species, and increased the activity of mitochondrial superoxide dismutase in tumor cells [228]. Since one of the biggest challenges today is certainly the treatment of malignant tumors, the fact that carnosine may have a role in this challenge is supported by the studies listed in Table 7. It is known that cancer cells have a high dependence on glycolysis for their production of adenosine triphosphate (ATP), while carnosine has the ability to inhibit glycolysis and thus achieve an antitumor effect [229,230]. Hipkiss et al. [231] discussed the potential mechanisms through which carnosine acts to inhibit glycolysis, and these are: influence on glycolytic enzymes, metabolic regulatory activities, redox biology, protein glycation, glycoxalase activity, apoptosis, gene expression, and metastasis. Carnosine inhibits glioblastoma growth, but independent of PI3K/Akt/mTOR signaling with a significant reduction of Akt phosphorylation in the U87 glioblastoma cell line. In addition, cell viability was reduced only in the presence of carnosine [232]. Carnosine has an effect in bladder cancer by stopping the G1 phase cell cycle by increasing p21WAF1 expression and decreasing cyclin/CDK complexes. Furthermore, it has an effect on promoter-matrix metalloproteinase-9 (MMP-9), which is a key regulator and participates in the metastasis of bladder cancer cells. Carnosine suppresses the binding of transcription factors NF-κB, Sp-1, and AP-1 to the MMP-9 promoter. In addition, it inhibits angiogenesis by suppressing VEGFR-2-mediated signaling pathways such as extracellular signal-regulatedprotein kinase (ERK), AKT, and nitric oxide synthase (eNOS) [233]. Carnosine inhibits metastatic cell adhesion and extravasation by suppressing nuclear factor kB (NF-κB) signaling pathway activation in human colon cancer cells and umbilical vein cells [234]. Carnosine decreased expression of hypoxia inducible factor 1 alpha (HIF-1α) in human colon cancer cells, which is a major cause of resistance to drugs. Furthermore, in combination with 5-fluorouracil (5-FU), it lowers the expression of some chemoresistance markers [235,236]. Carnosine retards senescence of human peritoneal mesothelial cells and inhibits progression of ovarian cancer cells, probably by inhibiting oxidative stress. Taken together, for these beneficial effects of carnosine, further studies to elucidate the exact mechanisms of its action are justified [237].

### 7.5. Carnosine and Vascular Function

Nitric oxide (NO) is a key inter- and intracellular mediator that plays an important role in the maintenance of vascular tone, platelet aggregation, and endothelial viability [238]. Different studies have shown a contradictory effect of carnosine on NO release and activity. While some studies indicate a stimulating effect of carnosine on NO formation, the results of some other studies are quite the opposite, indicating the inhibitory effect. For example, carnosine, in the presence of NADPH, stimulated activity of NO synthase as measured by soluble guanylate cyclase [239]. In cultured endothelial cells [240] carnosine facilitates NO production at concentrations higher than 5 mM, and the mechanisms may include a carnosine-induced rise in free calcium levels.

On the other hand, carnosine exhibited an inhibitory effect on NO-dependent activation of guanylate cyclase [241], which is attributed to NO interaction with the Fe-heme prosthetic group, suggesting that carnosine could interact directly with NO. Nicoletti at el. demonstrated a direct interaction of carnosine with NO and a correlation was found between cellular protection and NO free radical scavenging activity of carnosine in primary astroglia cell cultures [242].

NO production induced by dietary carnosine may locally dilate blood vessels, but may not reduce systemic blood pressure since reduced blood pressure in rats was noticed after intravenously administered a high dose (33.3 mg/kg) of carnosine. Furthermore, carnosine causes endothelium-independent vasodilation in isolated rat aorta, which emphasizes its functional importance in vasodilation despite damaged endothelium. These results are the opposite of those highlighted by O’Dowd et al. [243], demonstrating vasoconstrictive effect of carnosine on rabbit saphenous vein, suggesting a diverse effect of carnosine on endothelial cells in different blood vessels. While carnosine causes vasodilation of aortas, at least in part, through increased production of cGMP [244] (since guanylate cyclase inhibition by methylene blue significantly decreases it), it is suggested that a serotonin receptor may mediate vasoconstriction [245]. Similar receptors are lacking in the aorta.

In conclusion, carnosine may express both inhibitory and stimulatory effect on NO production and metabolism depending on the vascular bed, and it is still unclear under what conditions which mechanism is activated. Carnosine may also have important vascular features under conditions in which carnosine may be driven from tissue where its concentration is high (e.g., from muscle during exercise). Furthermore, carnosine supplementation or pharmacologic inhibition of carnosinase could be an efficient approach for blood pressure reduction in the treatment of hypertension [244].

Evidence from animal studies showed that carnosine supplementation may reduce body weight, blood pressure level, serum lipid levels, and atherosclerotic plaque instability, thus inhibiting the development and/or progression of hypertension and atherosclerosis [110,135,174,246,247]. However, well controlled human clinical trials investigating the role of carnosine in preserving and improving cardiovascular health are still scarce. A randomized placebo-controlled trial reported that 4-month treatment with a dietary supplement containing cinnamon, chromium, and carnosine (1.2 g/day) decreased fasting blood glucose and increased fat-free mass in overweight or obese pre-diabetic subjects [248]. Furthermore, other randomized controlled trial demonstrated that L-carnosine (500 mg/d), added to conventional therapy for six months, significantly improved cardiopulmonary exercise test and the 6-min walking test, but not left-ventricular ejection fraction in patients with chronic heart failure [188]. The fact that endothelial dysfunction, characterized by a loss of vasodilator and anti-inflammatory properties of endothelium, presents one of the earliest events in the pathological development of cardiovascular diseases [249], leads to the question of whether carnosine supplementation has the potential to modulate endothelium and vascular function, respectively. Such potential interaction between carnosine supplementation and vascular endothelium-dependent responses is still under investigated in both patients and healthy population. Evidence from a limited number of studies (mostly in animals) suggest that the potential of carnosine supplementation in improving and enhancing the cardiometabolic health of cardiovascular and diabetic patients lies in its anti-inflammatory and anti-oxidative properties [102,135,201]. However, a literature search that included the effect of carnosine (an over-the-counter food supplement) on endothelial function in humans did not show any study examining this issue. There is only a published protocol (but still not published results) for a randomized, double-blind, placebo-controlled trial on the effect of carnosine on cardiometabolic health in patients with pre-diabetes and type 2 diabetes, which will include endothelial function assessment by using non-invasive peripheral arterial tomography [146]. Therefore, it is expected that well-designed and controlled human clinical trials with large sample sizes will include functional assessment of vascular reactivity and endothelial function will provide valuable insights on the effect of carnosine on vasculature and endothelium in both cardiovascular patients (and/or population with increased cardiovascular risk) and healthy populations.

## 8. Conclusions

The objective of this review was to provide a state-of-the-art overview of the science of carnosine’s role in health and disease. As an endogenous substance, carnosine is easily absorbed by the digestive tract and can cross the blood–brain barrier [250,251]. Importantly, the equilibrium between carnosine and its constituent amino acids in vascular tissue may represent a new and novel mechanism for the modulation of vascular tone and function related to cardiovascular diseases. Since increased oxidative stress level and endothelial inflammation and activation (endothelium–leukocyte interaction) are the culprit of endothelial dysfunction development in various cardiovascular diseases [184,252], it is reasonable to assume that carnosine supplementation could have a beneficial effect on endothelial function, which would be manifested in part as improved vascular reactivity in both the macro- and microcirculation. Thus, investigation of its antioxidative properties in cardiometabolic diseases seems to be the major direction for forthcoming research activity, particularly in the form of dietary intake such as in functional food.

## Figures and Tables

**Table 1:** Extraction of carnosine from human samples.

Sample	Deproteinization	Extraction Method	Separation Media	pH	Ref.
muscle	perchloric acid	SPE	borate buffer	9.6	[32,33]
muscle	n.a.	Liquid.	borate buffer	9.6	[34]
muscle	n.a.	Liquid	water	n.a.	[35]
muscle	70% ethanol	Liquid.	water/NaOH, HCl	n.a.	[36]
muscle	35% sulfosalicylic acid	Liquid.	borate buffer	n.a.	[37,38]
muscle	acetonitrile	Liquid.	hydrochloric acid	n.a.	[39]
human plasma	35% sulfosalicylic acid	Liquid.	borate buffer	n.a.	[28,37]
human plasma	35% sulfosalicylic acid	Liquid.	PBS buffer	n.a.	[18]
human plasma	methanol	Liquid	water/formic acid	n.a.	[40]
human plasma/urine	EDTA	Liquid	sodium acetate buffer	6.4	[41]

**Table 2:** HPLC analysis.

Protocol	Column	Detection	Derivatization Reagent	Sample Matrix	Limits of Detection	Ref.
RP-HPLC	Hypersil ODS	Fluorescent	3-mercaptopropionic acid/*o*-phthaldehyde	muscle	0.005 mmol/kg	[32,33]
RP-HPLC	APEX ODS	Fluorescent	3-mercaptopropionic acid/*o*-phthaldehyde	muscle	0.005 mmol/kg	[34]
RP HPLC	Hypersil ODS	Fluorescent	3-mercaptopropionic acid/*o*-phthaldehyde	muscle	0.005 mmol/kg	[35]
JLC-300	LCR-6	n.a	n.a.	muscle	0.005 mmol/kg	[36]
RP HPLC	XBridge BEH	Fluorescent	AccQTag	muscle	n.a.	[38]
RP HPLC	Hypersilica	UV (210 nm)	n.a.	muscle	3 and 10 μM	[41]
RP HPLC	HILIC silica	UV (214 nm)	n.a	muscle		[39]
RP HPLC	Hypersil ODS	Fluorescent	3-mercaptopropionic acid/*o*-phthaldehyde	human plasma		[18]
UPLC	Acquity Peptide BEH C18	MS	n.a.	human plasma		[40]
RP HPLC	Jupiter C18	Fluorescent	carbazole-9-carbonyl chloride (CFC)	human serum, animal feed	15 nM	[28]

**Table 3:** Effect of treatments on the concentration of carnosine (mg/kg) in breasts and thigh muscles of broilers.

	Treatments	Content of Carnosine	
		Breast	Thigh
Kralik et.al. (2015) [160]	β-alanine 0.0%	756.15	-
	L-histidine 0.0%	941.58	-
	β-alanine 0.5%	753.29	-
	L-histidine 0.3%	1025.22	-
	β-alanine 1.0%	911.01	-
	L-histidine 0.5%	1186.06	-
Kralik et. al. (2018) [168]	Control	665.47	261.19
	β-alanine 0.5% + MgO 0.24%	715.45	420.64
	L-histidine 0.25% + MgO 0.24%	736.17	467.40
	β-alanine 0.20% + L-histidine 0.10% + MgO 0.24%	1084.25	495.01

**Table 4:** The effects of carnosine in animal models of metabolic syndrome or type 2 diabetes mellitus.

Study Design	Daily Intake of Carnosine	Main Findings of Carnosine Effects
Rats (n = 40) received conventional diet (control), high-fat high-carbohydrate diet, carnosine and conventional diet, or carnosine and high-fat high-carbohydrate diet [175]	250 mg/kg/daily intraperitoneal; 16 weeks	-reduced abdominal obesity, blood pressure, glucose; -normalized total cholesterol, LDL level -no effect on insulin, leptin and adiponectin concentrations
BTBR (Black and Tan, BRachyuric) ob/ob mice (n = 35), a type 2 diabetes model with a phenotype like advanced human diabetes nephropathy [176]	45 mg/kg body weight dissolved in drinking water	-improved glucose metabolism, albuminuria and restored the glomerular ultrastructure
Effect of carnosine on renal function, oxidation and glycation products in the kidneys of high-fat diet/streptozotocin-induced diabetic rats (n = 24) [179]	250 mg/kg body weight; intraperitoneal, 5 times a week; 4 weeks	-decreased serum lipids, creatinine, and urea levels, oxidation products of lipids and proteins, advanced glycation end products (AGEs) levels

**Table 5:** The effects of carnosine in patients with metabolic syndrome or type 2 diabetes mellitus.

Study Design	Daily Intake of Carnosine	Main Findings of Carnosine Effects
Obese type 2 diabetic patients (n = 82) were either supplemented daily with α-lipoic acid, carnosine and thiamine [181]	7 mg α-lipoic acid/kg body weight, 6 mg carnosine/kg body weight, and 1 mg thiamine/kg body weight or placebo for 8 weeks	-reduced glucose and HbA1c levels, probably by increasing insulin production from pancreas
Patients with type 2 diabetes mellitus (n = 54) divided into two groups, received either placebo or carnosine [129]	L carnosine 2 capsules of 500 mg each for 12 weeks.	-decrease in fat mass, fasting blood glucose, glycated hemoglobin and serum levels of triglycerides

**Table 6:** The effects of carnosine in cardiovascular diseases.

Study Design	Daily Intake of Carnosine/Cells Exposure with Carnosine	Main Findings of Carnosine Effects
Carnosine was tested for its ability to counteract oxidative stress in macrophages [186]	Carnosine (5, 10, 20 mM)	-multimodal mechanism of action on macrophage cells under oxidative stress and inflammation conditions
Mitochondria from myocardial atrial samples were isolated and incubated for 3 h at 37 °C with 75-µM norepinephrine NE) and increasing concentrations of carnosine (1, 2.5, 5, 10, and 25 mM) [187]	Carnosine (1 mM)	-carnosine block formation of catecholaldehyde protein adducts in isolated human cardiac mitochondria treated with NE
Rats (n = 24) were treated with carnosine or carnosine + vitamin E. On the 8th day of treatment, rats were injected with a single dose of doxorubicin [189]	Carnosine 250 mg/kg/day i.p. or carnosine + vit E (200 mg/kg) once every 3 days; i.m.); for 12 days	-carnosine and especially in combination with vitamin E, protect against doxorubicin-induced toxicity in heart, liver, and kidney tissues of rats
Patients (n = 50) with stable chronic heart failure (CHF) and severe left-ventricular systolic dysfunction on optimal medical therapy were randomized 1:1 to receive oral or dispersible carnosine or standard treatment [188]	Carnosine 500 mg once a day; 6 months	-beneficial effects on exercise performance and quality of life in stable CHF

**Table 7:** Overview of studies of the mechanism of action of carnosine in malignant diseases.

Study Design	Tumors	Cells Exposure with Carnosine	Main Findings of Carnosine Effects
Experimental Two cell lines (U87 and T98 G) [232]	Glioblastoma	Carnosine (50 mM), the PI3K inhibitor Ly-294,002 (5 μM), the mTORC1 inhibitor rapamycin (25 nM) and combinations of the compounds for 24 h	-reduces Akt phosphorylation in the U87 cell line
Experimental In EJ bladder cancer cells and EJ-xenografted BALB/c nude mice (n = 7 per each group) [233]	Bladder cancer	Carnosine (0,10, 20, 50, and 100 mM) for 24 h. Carnosine (0, 5, 10 mg/kg) were administered by oral gavage daily. Then the efficacy was compared with that of 5 mg/kg cisplatin, the positive control.	-stops the G1 phase cell cycle, suppresses the binding of transcription factors on the MMP-9 promoter -in xenograft tumors exhibited antitumor activity equivalent to cisplatin, but no weight loss occurred in carnosine-treated mice.
Experimental The HCT-116 human colorectal cancer cell line The EA.hy926 human umbilical vein endothelial cell line Cells treated with sterile water served as the control [235,236]	Colorectal cancer	Carnosine (0.5, 1 or 5 mM) was added EA.hy926 cells for 12, 24, 48 h. HCT-116 cells were treated with 1 μg/mL lipopolysaccharide, then 0.5, 1 or 5 mM carnosine combined with 1 μg/mL LPS was added and cells were incubated for 24 h	-suppress adhesion of HCT-116 cells to EA.hy926 cells and extravasation. -inhibits the NF-kB signaling pathway activation -reduced the permeability of EA.hy926 cell–cell junctions -inhibited HCT-116 cell adhesion to EA.hy926 cells
Experimental Primary human peritoneal mesothelial cells, three ovarian cancer cell lines: A2780, OVCAR-3 and SKOV-3 [237]	Ovarian cancer	L-carnosine (20 mM)	-inhibits mitochondria-related oxidative stress

## Data Availability

Data sharing not applicable.
